# Determinant of Mother's Health Promotional Measures Practice of Infant with Age 6–12 Months in a Tertiary Hospital of Nepal

**DOI:** 10.1155/2021/6647230

**Published:** 2021-06-10

**Authors:** Chet Kant Bhusal

**Affiliations:** Department of Community Medicine, Universal College of Medical Science and Teaching Hospital, Tribhuvan University, Bhairahawa, Rupandehi, Nepal

## Abstract

**Background:**

Promotion of health is vital for the optimal growth and development of every infant. Globally, many infants died due to common problems such as diarrhoea and respiratory infection; most of these problems are related to inadequate breast feeding, improper complimentary feeding, lack of immunization, and home accident. Infant and child health status depends upon mothers' knowledge and practice regarding health promotional measures. This study aimed to determine practice and its determinants regarding health promotional measures of infant with 6–12 months age amongst the mothers attending Pediatrics Outpatient Department of Universal College of Medical Science and Teaching Hospital, Bhairahawa, Rupandehi, Nepal.

**Methods:**

Hospital-based cross-sectional study was conducted in Universal College of Medical Science, Bhairahawa, Rupandehi, Nepal, among 414 mothers attending pediatrics outpatient department from September 2019–March 2020. Purposive sampling technique was used to select mothers of infants aged 6–12 months. Bivariate analysis was used primarily to assess the association between dependent and independent variables. Variables which were associated in bivariate analysis with *p* < 0.05 were entered into a multivariable logistic regression model to identify associated factors of health promotional measures. The goodness of fit of multivariate logistic regression was checked by Nagelkerke *R* square and variation inflation factor.

**Results:**

The mean age and family size was 25.89 ± 4.81 years and 5.94 ± 2.48, respectively. A total of 71.5% mothers have good practice of health promotional measures. Mothers from Dalit caste (adjusted odds ratio = 0.04, confidence interval: 0.005–0.30), mothers with below school leaving certificate education (AOR = 0.08, CI: 0.02–0.27), fathers engaged in nonagricultural work (AOR = 7.21, CI: 2.59–20.11), birth space of index child greater than 2 years (AOR = 12.88, CI: 3.49–47.58), and family monthly income greater than 20000 Nepalese rupees (AOR = 3.29, CI: 1.16–13.32 were significantly associated with good practice of health promotional measures.

**Conclusions:**

More than one-fourth of the mothers have poor practice of health promotional measures. Ethnicity, mothers' education, fathers' occupation, birth space of index child, and family monthly income were found to be independent determinants of practice of health promotion measures. Thus, policy makers should provide specific education regarding health promotional measures to both parents.

## 1. Introduction

The infancy is period when child attain rapid physical, social growth, and development. Promotion of optimum health of infancy includes nutrition, growth monitoring, sleep and activity, prevention of infection, accidents, play, and stimulation and anticipatory guidance [1]. The infants are dependent and susceptible for permanent effects of risky and vulnerable activities; hence, the need of health maintenance has greater importance [2]. Health promotion is the process of enabling people to increase control over and to improve their health [3]. Mother is the significant person for the promotion of psychosocial development of infants and children. Healthcare providers should assist the parents to make decisions and to take care of their children according to their individual child's needs [4]. Maternal knowledge regarding child development has influence on the way mothers act together with their offspring [5]. The major components of the health promotion of infant are breastfeeding, complementary feeding, immunization, and accident [1]. Breastfeeding is widely regarded as the best form of infant feeding, with many advantages for both the infants and the mothers [6]. Exclusive breastfeeding is characterized as a mother who breastfeeds her child exclusively for six months without supplementation, not even water with exception of oral rehydration solution, drops, or syrups consisting of vitamins, minerals supplements, or medicines [7]. Reduced morbidity related to gastrointestinal illness is a significant benefit with exclusive breastfeeding from 4 to 6 months. Perhaps most notably, exclusive breastfeeding has been linked to a lower infant mortality rate [8]. The complementary feeding is a critical window for reducing stunting, wasting, overweight, and obesity, as well as optimizing long-term growth and health [9]. Immunization allows for the promotion of coordinated care and the improvement of recipients' overall health and wellbeing [10]. Children's accidents at home are rapidly being recognized as a public health issue that could be avoided with greater awareness, good practices, and changes to the home climate [11].

Evidence shows that only about 35% of infants worldwide are exclusively breastfed during the first four months of life; complementary feeding frequently begins too early or too late, and foods are often nutritionally inadequate and unsafe [12]. A study of infant feeding practices of mothers in an urban area of Pokhara Nepal revealed that only 43.5% of the mothers initiated breastfeeding within one hour of birth and 60.5% were practicing exclusive breastfeeding at 5 months [13].

Poor quality complementary foods with low-nutrient density and inappropriate feeding practices have been identified among the major causes of malnutrition in young children. In many developing countries, complementary foods are introduced too early or too late, and the quality and quantity of the foods are insufficient, leading to a great risk of nutritional deficiencies during the second half of infancy [14]. Meeting minimum standards of dietary quality is a challenge in Nepal. Children were not receiving complementary foods at the right age (either too early or too late), were not fed frequently enough, and the quality of the food might be inadequate [15]. Infant mortality rate (IMR) of the world is 36.58/1000 live births [16]. Globally, 5.2 million children under the age of 5 years and 2.4 million newborns died in the first month of life in 2019 [17]. More than half of these early child deaths are due to conditions that could be prevented or treated with access to simple, affordable interventions like vaccination. Though more than 1 billion children vaccinated over the last decade, global vaccination coverage has stuck at 85% with no significant changes during the past year [18]. The most common causes of death of the infants are diarrhoea and respiratory infection. The problems are related to inadequate breast feeding, lack of knowledge in complimentary feeding, and lack of immunization. The other most common cause of death is home accident. Mothers can prevent or minimize these problems by proper care of infants [16].

In order to maintain the proper health status of infant, proper infant health promotion is necessary. Mother has main parenting role of her infant rather than father, as she spends almost all time with her infant. Health promotion of infant depends upon the level of practice of mother. Although growing literatures are found on various aspects of breast feeding, complementary feeding, immunization, and prevention of accidents among infants, there are very few studies carried out to investigate the determinants of practice of health promotional measures of infant among mothers. Therefore, this study was conducted to identify the practice and different determinants affecting practices of health promotional measures of infant with 6–12 months age among the mothers attending Pediatrics Outpatient Department (OPD) of Universal College of Medical Science and Teaching Hospital (UCMS-TH), Bhairahawa, Rupandehi, Nepal.

## 2. Materials and Methods

### 2.1. Study Design and Source of Population

Hospital-based cross-sectional study was conducted in Universal College of Medical Science, Bhairahawa, Rupandehi, Nepal, from September 2019–March 2020. Mothers having child aged six to twelve months attending pediatrics OPD of UCMS were included in the study. Similarly, mothers with severe mental problems who have no willingness to participate were excluded from the study.

### 2.2. Sample Size Determination and Sampling Technique

The sample size of the study was 414 which was calculated by using formula *N* = *Z*^2^*pq*/*L*^2^ with 95% level of confidence interval, critical value *Z* = 1.96, 5% margin of error, and 56.81% mothers fed their children complementary food of appropriate consistency [19]. Hence, *N* = *Z*^2^*pq*/*d*^2^ = (1.96)^2^ × (0.57) × (0.43)/(0.05)^2^ = 376. Adding 10% nonresponse rate, the final sample size (*n*) = 414. A nonprobability purposive sampling technique was used to select the sample. Those respondents who were attending pediatrics OPD of Universal College of Medical Science and Teaching Hospital were judged at first whether they can fulfil the inclusion criteria, and they were selected purposively after taking written informed consent.

### 2.3. Data Collection Procedures and Validity

A set of semistructured questionnaire was formulated as data collection tools to collect the information of the respondents. Initially, the questionnaire was prepared in English and then translated in to Nepali language. Nepali questionnaire was retranslated to English to find consistency of question. Pretesting of the questionnaire was done among 10% of the sample in nearby private clinic of Bhairahawa, Nepal. Two-day training was provided to four pediatric residence doctors and one final year bachelor in public health student of UCMS. Face-to-face interview was conducted among selected mothers. All the filled questionnaires were reviewed and checked by the principal investigator on regular basis.

### 2.4. Data Processing and Analysis

Collected data were followed by a cross-check to ensure all the questions have been filled correctly. Data checking, compiling, and editing were carried out manually by the researcher. Collected data were entered into Microsoft Excel, and SPSS software version 22 was used to analyze the data. Simple frequency tables, cross tables, and mean tables had been used to present data related to the study. Relationship of the practice level regarding health promotional measures with different independent variables was compared and tested using the chi-square test, and significance will be considered when *p* < 0.05. Furthermore, crude odds ratio and confidence interval were calculated using binary logistic regression. Those variables which were found significantly associated in binary logistic regression with dependent variables were entered into the multivariate logistic regression model to find the adjusted odds ratio and confidence interval for the final determinants of health promotional measures of infants. The goodness of fit of multivariate logistic regression was checked by Nagelkerke *R* square, and multicollinearity was checked by the variation inflation factor. The value of Nagelkerke *R* square was 0.449; which showed that goodness of fit is adequate. Similarly, VIF of all independent variables was less than 10 which lies in the range 1.01–2.04, so multicollinearity was not available among independent variables. After testing this, multivariate logistic regression was calculated to find the net effect of independent variables in mother's health promotional measures practice of infants with the 6–12 months age group. Then, adjusted odds ratio (AORs) was estimated, and their corresponding value at 95% confidence intervals was calculated.

### 2.5. Setting

The present study was conducted in Universal College of Medical Science and Teaching Hospital, Bhairahawa, Rupandehi district of Nepal. Rupandehi district of Lumbini Zone comes under the western development region. Rupandehi district is situated in the lovely lap of the Chure range and surrounded by Palpa on the north, India on the south, Kapilvastu on the west, and Nawalparasi on the east. The total area of this district is 1172 square kilometer. The geographical position of the district is 830 10″ to 830 30″ longitudes in the east and 270 10″ to 270 45″ latitude in the north. The total surface area of the district is 141,340 ha with an altitude ranging from 95 m to 1219 m above the sea level [20]. According to CBS 2012, district has total population of 8,80,196 [21].

Universal College of Medical Sciences (UCMS) is a tertiary level hospital, established in the year 1998 with the affiliation of T.U and recognized by Nepal Medical Council (NMC), and considered as the pioneer institute in Nepal in the field of medicine and dentistry.

### 2.6. Measurement of Practice regarding Health Promotional Measures

Health promotion measures in this study referred to those acts or activities of the mother which promote the health of infant which comprises of exclusive breastfeeding, complimentary feeding, immunization, and prevention of accident. There were 20 questions related to practice regarding health promotional measure of infant. It consisted of four subscales which comprised of practice regarding breastfeeding, practice regarding complementary feeding, practice regarding immunization, and practice regarding prevention of accidents each consisting of 7, 6, 3, and 4 items, respectively. In the each subscale, single response items were only included. So the score in each subscale was 7, 6, 3, and 4, respectively. The total score of this section was 20. The practice score was categorized as good practice, ≥50% of total score, and poor practice, <50% total score.

### 2.7. Ethical Consideration and Informed Consent

This study received ethical approval from Institutional Ethical Review Board of Universal College of Medical Science and Teaching Hospital, Bhairahawa, Rupandehi, Nepal (UCMS/IRC/126/19). Moreover, all the participants were fully informed about the nature of study, research objectives, privacy, and confidentiality of the collected information. Prior to interview, written informed consent was taken from the respondents. Only those respondents who voluntarily agree to participate were involved in the study. All study participants were informed of their right to refuse participation and to leave the interview at any time. The researcher had followed the principals of justice, human dignity, and physical wellbeing of the respondents. The respondent and their thoughts were respected.

## 3. Results

The mean age and family size was 25.89 ± 4.81 years and 5.94 ± 2.48, respectively. More than half (53.9%) of respondents had nuclear family. Four-fifths (80.2%) of the respondents were Hindu. More than four-fifths (89.1%) of the respondents infant lived with both mothers and father in family. Nearly two-thirds (64.7%) of the respondent had two or less than two living children. More than two-thirds (69.8%) of the respondents' family earned less than or equal to twenty thousand Nepalese rupees (NRs) per month. Nearly one-fourth (23.7%) of the infants' mothers had received education of secondary and above intermediate levels. About half (49.3%) of the infants' father had received education of intermediate and above levels. About half (49.5%) of infants' mothers were housewives. About two-fifths (39.4%) of infants' father were engaged in small scale business (Table 1).

Regarding practice of breast feeding more than four-fifths (87.4%) of the infant's mother have fed colostrum to their babies, whereas nearly one-fifth (19.6%) of the infant's mother have fed anything else than breastfeeding after birth. Likewise, regarding complementary feeding, most (95.2%) of the infant's mother started complementary feeding with lito, mashed cereals, and fruits; similarly, very less (10.6%) of the infant's mother have fed at least three to four items of food at a time. Majority (93.2%) of the infant's mother had immunized their babies as per national immunization schedule and more than half (55.6%) of the infant's mother had taken their babies to hospital when fever arise after vaccination. About three-fourths (76.3%) of the infant's mother avoid small piece of toys and other things in order to prevent from ingestion injury, and similarly, more than one-fourth (33.3%) of the infant's mother does not leave child alone to prevent them from home accidents (fall injury) (Table 2).

About three-fourths (71.5%) of the mothers' of infant have good practice regarding health promotional measure (Table 3).

Table 4 provides sociodemographic, socioeconomic, and child-related factors associated with the practice level of health promotional measures. Variables such as ethnicity, religion, mother's education, father's education, father's occupation, birth space of index child, and family monthly income were found statistical significant with *p* value lesser than equal to 0.05 in bivariate analysis, and these variables were entered into the multivariate logistic regression analysis model which identified ethnicity, mother's education, father's occupation, birth space of index child, and family monthly income as associated factors with the practice level of health promotional measures. Mothers who were from Dalit caste were 96% less likely (AOR = 0.04, CI = 0.005–0.30) to have good practice regarding health promotional measures than those who were from Brahmin/Chhetri caste. The odds of having good practice regarding health promotional measures were reduced by 92% (AOR = 0.08, CI = 0.02–0.27) among mothers having educational backgrounds below school leaving certificate (SLC) than their counterparts who were from illiterate backgrounds. Regarding the father occupation, the odds of having good health promotional measures were 7.21 more likely (AOR = 7.21, CI = 2.59–20.11) among fathers who were engaged in other than agriculture work than those who were involved in agriculture work. Mothers whose index child had birth space of greater than 2 years were 12.88 times more likely (AOR = 12.88, CI = 3.49–47.58) to have good practice of health promotional measures than their counterparts. Similarly, those respondents whose monthly family income was greater than NRs 20000 were 3.29 times more likely (AOR = 3.29, CI = 1.16–13.32) to have good practice regarding health promotional measures than those whose family income was lesser than equal to NRs 20000 (Table 4).

## 4. Discussion

Ethnicity, mother's education, father's occupation, birth space of index child, and family monthly income were associated factors of good practice of health promotional measures in this study. Mothers from Dalit caste have poor practice on health promotional measures; this might be due to that Dalit is lower caste and Nepalese society is higher caste dominant society. Mothers having below SLC level education have poor practice regarding health promotional measures. Likewise, fathers who were engaged in nonagricultural work have practiced good health promotional measures.

More than two-thirds of the infant's mothers had good level of practice regarding health promotional measures in this study; however, in contrast to this study, another study conducted in Nepal found that less than one-third of the mothers had good level of practice [22]. This disparity might be due to the divergence scoring system for measuring the health promotional measures.

More than three-fourth of the infants in the current study were fed with breast milk during the survey; however, lower proportion of infants with 6–12 months were fed with breastfeeding in the study conducted in Italy [23]. This difference might be due to different study setting and methodology adopted by two different studies. The present study found slightly less than three-fourths of the babies were exclusively breast fed (EBF) for complete 6 months of age. However, several other studies conducted in different parts of the Nepal and other countries such as Nepal Demographic and Health Survey [24], and other studies conducted in Nepal [25–27], south India [28], Trongsa District, Bhutan [29], northwest Ethiopia [30], and Jimma town, southwest Ethiopia [31] reported a lower proportion of the babies were exclusively breast fed for 6 months than the present study. In contrast to this study, another study conducted in Chepang communities of Makawanpur district, Nepal [32], and Northwest Ethiopia [33] found that higher proportion of the babies were exclusive breast fed until 6 months. These differences might be due to different study setting, methodology adopted, change in time, and advancement of educational materials as compared to previous time. Nearly about one-fifth of the mothers fed prelacteal after their baby birth which is in line with another study conducted in Bhaktapur, Nepal, [34] and rural Bangladesh [35]. However, in contrast to this study, other studies such as studies carried out in Nepal [36, 37], Kinshasa, DR Congo [38], and Nairobi, Kenya [39], found higher proportion of mothers give supplementation prior to breast feeding initiation. This difference might be due to different study setting and level of awareness among respondents. Majority (87.4%) of the mothers in this study fed colostrum to their baby, which is in line with another national study conducted in Nepal [40], in Bhaktapur, Nepal [34], and in India [41]. More than half of the respondents in this study initiated breastfeeding within the first hour after birth, which is in line with the national level data of Nepal demographic and Health Survey 2016 [24] and another study conducted in Nepal [34]. However, another study conducted in western Nepal found that lower portion of mothers initiated their breast feeding to their babies within an hour [42]. This divergence might be due to the level of respondents as previous study was carried out in slum area. Less than three-fifths of the mothers in this study did burping to their babies after feeding breast milk; however, in contrast to this study, another study conducted in India found three-fourths of the mothers practiced burping after feeding breast milk to their babies [43]. This difference might be due to different study setting and methodology adopted in two different studies.

The present study revealed that around three-fifths of the mothers started complementary feeding after 6 months which is in line with the study conducted in Nepal [21, 44], south India [45], and Ethiopia [46]. However, in contrast to this study, another study conducted in Nepal found only around one-fifth and study conducted in Kosova [47] found around two-fifths of the mother initiated complementary feeding after 6 months of age [48]. This divergence might be due to the reason that previous study was conducted in community setting and this study is in hospital setting. Majority of the respondents in the present study continued feeding their breast milk along with complimentary feeding to their children which is in line with another cross-sectional study conducted in Chepang communities of Nepal [32]. The present study revealed that more than three-fifths of the respondents washed their hand with soap and water before giving food to their infants; however, in contrast to this study, another study conducted in Singapore found lower portion of the mothers washed their hands before feeding their babies [49]. This divergence might be due to difference in study setting and level of community respondents in the previous study.

In this study, majority of the respondents immunized their babies as per National Immunization Schedule; however, the national level survey reported lower percentage of children aged 12–23 months had received all basic vaccinations [24]. The national data suggested high dropout rates have been recorded in the Terai area, which has a high population density [21]. This divergence might be due to age differences of children. More than four-fifths of the respondents took their babies to hospital during any side effect that arises after vaccination; however, in contrast to this study, another study conducted in Kathmandu, Nepal, found that lower proportion of the respondents took their babies to hospital [22]. This might be due higher education level of the respondents in this study.

The present study revealed that one-third of the respondents did not leave their infant alone to prevent from home accidents (fall injury) which is not consistent with another study conducted in Bara district, Nepal, where large portion of mothers do not leave their small child alone or without guarding [50]. This might be due to different study setting in these two studies. More than three-fourths of the respondents in this study avoid small piece of toys and other things in order to prevent their children from ingestion injury; however, another study conducted in Kathmandu, Nepal, found lower proportion of the mothers avoid such things to prevent their children from ingestion injury [22]. This might be due to the different study setting and higher education level of the respondents in this study.

The present study revealed that mothers' ethnicity was significantly associated with the practice of health promotional measures which is in line with the study conducted in Nairobi, Kenya, where mothers' ethnicity was significantly associated with suboptimal infant breastfeeding and feeding practices [39]. Mothers' education in this study was significantly associated with practice of health promotional measures which is in accordance with the study conducted in Chepang communities in Nepal [32] where mothers education was associated with infant and young child feeding, in Ethiopia [51] where mother education is significantly associated with exclusive breastfeeding practice, in Nairobi, Kenya [39] where suboptimal infant breastfeeding and feeding practices was significantly associated with mothers education. Fathers' occupation in this study was significantly associated with practice of health promotional measures which is in accordance with several other studies conducted in Ethiopia [46, 52] where fathers' occupation was positively associated with appropriate weaning practice of infants. Family' monthly income in this study was significantly associated with practice of health promotional measures which is in agreement with the study conducted in Bara, Nepal [50], where exclusive breastfeeding and prevention of childhood accident was significantly higher among richest household.

### 4.1. Strengths and Limitations of the Study

The strength of this study is it to provide the aggregate result of mother's health promotional measures including various components such as breastfeeding, especially exclusive breastfeeding, complementary feeding, immunization, and home accident of infants with age group 6–12 months. The limitation of the study is that it was conducted in only one tertiary hospital of Nepal, and purposive sampling was used as sampling technique.

## 5. Conclusions

More than one-fourth of the mothers have poor practice of health promotional measures. Ethnicity, mothers' education, fathers' occupation, birth space of index child, and family' monthly income were found to be independent determinants of practice of health promotion measures. In this context, it is recommended that administrators and policy makers should provide specific education regarding health promotional measures including breast feeding, complementary feeding, immunization, and prevention of accidents to both the parents focusing on rural area.

## Figures and Tables

**Table 1:** Background related characteristics of mothers of infant with age group 6–12 months in a tertiary hospital of Nepal.

General characteristics	Frequency (*n* = 414)	Percentage
Caste/ethnicity		
Brahmin/Chhetri	145	35.0
Madeshi	89	21.5
Dalits	28	6.8
Newar	23	5.6
Janjati	59	14.3
Muslim	70	16.9

Religion		
Hindu	332	80.2
Non-Hindu (Christian and Muslim)	82	19.8

Types of family		
Nuclear	223	53.9
Joint or extended	191	46.1

Education of mother		
Illiterate and informal class	81	19.6
Primary	44	10.6
Secondary	98	23.7
SLC	93	22.5
Intermediate and above	98	23.7

Education of father		
Illiterate and informal class	33	8.0
Primary	41	9.9
Secondary	63	15.2
SLC	73	17.6
Intermediate and above	204	49.3

Occupation of father		
Agriculture	87	21.0
Small scale business	163	39.4
Service (government and private)	134	32.4
Wage labor	14	3.4
Foreign labor	16	3.9

Birth space of index child (*n* = 357)		
≤2 years	272	65.7
>2 years	85	20.5

Earning status (cash money)		
Earning	352	85
No earning	62	15

Family monthly income in Nepalese rupees (NRs)		
≤20000	289	69.8
>20000	125	30.2

SLC, school leaving certificate.

**Table 2:** Mothers' practice regarding health promotional measure of infant with age group 6–12 months in a tertiary hospital of Nepal.

Practice regarding health promotional measures	Number (*n* = 414)	Percentage
Breast feeding		
Feeding anything else than breastfeeding after birth	81	19.6
Feeding colostrum to the baby	362	87.4
Breastfeeding within an hour of birth	223	53.9
Exclusive breastfeeding for complete 6 months	304	73.4
Recently feeding breast milk	323	78.0
Burping baby after feeding breast milk	232	56.0
Wiped breast with clean cloth before breast feeding	134	32.4

Complementary feeding		
Started complementary feeding after 6 months	244	58.9
Started complementary feeding with lito, mashed cereals, and fruits	394	95.2
Continuation of breast feeding with complementary feeding	366	88.4
Washing hands with soap and water before giving food	257	62.1
Feeding at least three to four items of food at a time	44	10.6
Feeding according to infant wish during their sickness	85	20.5

Immunization		
Immunized baby as per national immunization schedule	386	93.2
Taking baby to hospital when fever arise after vaccination	230	55.6
Taking baby to hospital in any side effect after vaccination	359	86.7

Prevention of accidents		
Keep burned part on water in home during child burn	224	54.1
Avoid small piece of toys and other things in order to prevent from ingestion injury	316	76.3
Does not leave infant alone to prevent from home accident (fall injury)	138	33.3
Some members supervised infant to prevent during playing	249	60.1

**Table 3:** Level of practice regarding mother's health promotional measures of infant with age group 6–12 months in a tertiary hospital of Nepal.

Characteristics	Frequency (*n* = 414)	Percentage
Poor practice	118	28.5
Good practice	296	71.5
Total	414	100.0

**Table 4:** Determinants of mother's practice level of health promotion measures of infant with age group 6–12 months in a tertiary hospital of Nepal.

Characteristics	Practice level (%)		*P* value	^a^COR 95% CI	^b^AOR 95% CI
	Poor practice	Good practice			
Ethnicity					
Brahmin/Chhetri	41 (28.3)	104 (71.7)		1	1
Madeshi	22 (24.7)	67 (75.3)	0.028^∗^	1.20 (0.66–2.19)	1.17 (0.52–2.63)
Dalits	8 (28.6)	20 (71.4)		0.99 (0.40–2.42)	0.04 (0.005–0.30)
Newar	4 (17.4)	19 (82.6)		1.87 (0.60–5.84)	1.24 (0.22–7.17)
Janjati	12 (20.3)	47 (79.7)		1.54 (0.74–3.20)	3.36 (0.87–12.99)
Muslim	31 (44.3)	39 (55.7)		0.50 (0.27–0.90)	0.14 (0.01–1.89)

Religion					
Hindu	82 (24.7)	250 (75.3)		1	1
Non-Hindu	36 (43.9)	46 (56.1)	0.001^∗^	0.42 (0.25–0.69)	5.35 (0.43–66.42)

Types of family					
Nuclear	66 (29.6)	157 (70.4)		1	—
Jointed and extended	52 (27.2)	139 (72.8)	0.594	1.12 (0.73–1.73)	

Mother's education					
Illiterate and informal	21 (25.9)	60 (74.1)		1	1
Below SLC	54 (38.0)	88 (62.0)		0.57 (0.31–1.04)	0.08 (0.02–0.27)
SLC and above	43 (22.5)	148 (77.5)	0.007^∗^	1.21 (0.66–2.20)	0.09 (0.02–0.37)

Father's education					
Illiterate and informal	18 (54.5)	15 (45.5)		1	1
Below SLC	40 (38.5)	64 (61.5)	<0.001^∗^	1.92 (0.87–4.23)	1.10 (0.25–4.85)
SLC and above	60 (21.7)	217 (78.3)		4.34 (2.07–9.12)	3.24 (0.70–14.91)

Mother's occupation					
Housewives	53 (25.9)	152 (74.1)		1	—
Other than housewives	65 (31.1)	144 (68.9)	0.237	0.77 (0.50–1.19)	

Father's occupation					
Agriculture	38 (43.7)	49 (56.3)		1	1
Other than agriculture	80 (24.5)	247 (75.5)	<0.001^∗^	2.39 (1.46–3.92)	7.21 (2.59–20.11)

Number of living children					
≤2 children	68 (25.4)	200 (74.6)	0.056	1	—
> children	50 (34.2)	96 (65.8)		0.65 (0.42–1.01)	

Birth space (index child)					
≤2 years	85 (31.3)	187 (68.8)		1	
>2 years	5 (5.9)	80 (94.1)	<0.001^∗^	7.27 (2.84–18.60)	12.88 (3.49–47.58)

Earning status					
Earning	94 (26.7)	258 (73.3)		1	—
No earning	24 (38.7)	38 (61.3)	0.054	0.58 (0.33–1.01)	

Family monthly income (NRs)					
Earning ≤ 20000	103 (35.6)	186 (64.4)		1	1
>20000	15 (12.0)	110 (88.0)	<0.001^∗^	4.061 (2.25–7.33)	3.29 (1.16–13.32)

^∗^Significant at *p* < 0.05, 1, reference category. ^a^Crude odds ratio. ^b^Adjusted odds ratio.

## Data Availability

The data used to support the findings of this study are available from the corresponding author upon request.

## Abbreviations

AOR: Adjusted odds ratio
CI: Confidence interval
OPD: Outpatient department
OR: Odds ratio
SD: Standard deviation
SLC: School leaving certificate
SPSS: Statistical Package for the Social Sciences
UCMS-TH: Universal College of Medical Science and Teaching Hospital.
